# The research on TBATS and ELM models for prediction of human brucellosis cases in mainland China: a time series study

**DOI:** 10.1186/s12879-022-07919-w

**Published:** 2022-12-12

**Authors:** Daren Zhao, Huiwu Zhang

**Affiliations:** Department of Medical Administration, Sichuan Provincial Orthopedics Hospital, Chengdu, Sichuan China

**Keywords:** Human brucellosis, ELM model, TBATS model, Time series, Prediction

## Abstract

**Background:**

Human brucellosis is a serious public health concern in China. The objective of this study is to develop a suitable model for forecasting human brucellosis cases in mainland China.

**Methods:**

Data on monthly human brucellosis cases from January 2012 to December 2021 in 31 provinces and municipalities in mainland China were obtained from the National Health Commission of the People’s Republic of China website. The TBATS and ELM models were constructed. The MAE, MSE, MAPE, and RMSE were calculated to evaluate the prediction performance of the two models.

**Results:**

The optimal TBATS model was TBATS (1, {0,0}, -, {< 12,4 >}) and the lowest AIC value was 1854.703. In the optimal TBATS model, {0,0} represents the ARIMA (0,0) model, {< 12,4 >} are the parameters of the seasonal periods and the corresponding number of Fourier terms, respectively, and the parameters of the Box-Cox transformation ω are 1. The optimal ELM model hidden layer number was 33 and the R-squared value was 0.89. The ELM model provided lower values of MAE, MSE, MAPE, and RMSE for both the fitting and forecasting performance.

**Conclusions:**

The results suggest that the forecasting performance of ELM model outperforms the TBATS model in predicting human brucellosis between January 2012 and December 2021 in mainland China. Forecasts of the ELM model can help provide early warnings and more effective prevention and control measures for human brucellosis in mainland China.

**Supplementary Information:**

The online version contains supplementary material available at 10.1186/s12879-022-07919-w.

## Background

Brucellosis is a globally important endemic zoonotic disease [1]. The disease is transmitted to people by direct or indirect contact with infected animals or their excrement, and by eating contaminated food or dairy products [2, 3]. The major clinical manifestations of brucellosis are fever, weakness, arthralgia, and sweating [4]. Brucellosis remains a major public health problem and poses a significant threat to human health and economic consequences [5]. Globally, brucellosis epidemics are mainly found in the Asian and European regions of low- and middle-income countries across the Mediterranean region [6, 7], Arabian Peninsula, Africa, Asia, and Central and South America, including approximately 170 countries and regions [8]. Moreover, human brucellosis affects 1/6 to 1/5 of the global population [8], leading to serious health consequences and depression in livestock development worldwide.

Human brucellosis remains a serious public health issue in China [9]. It is estimated that approximately 350 million people in China are affected by human brucellosis [8]. Although great progress in the prevention and control of human brucellosis has been achieved in China, the prevalence of this infectious disease remains relatively high level in China [10]. Therefore, prevention and control of human brucellosis remains one of the most important public health issues in China. Scientific prediction and analysis of human brucellosis can help provide proper policy and public health planning by governments and relevant departments. Early warning of brucellosis is crucial for the prevention and control of this infectious disease and can serve as a foundation for the allocation of healthcare resources in advance. Therefore, there is an urgent need to develop scientific and reliable forecasting techniques for brucellosis epidemic trend prediction.

Previous human brucellosis incidence predictive modeling studies can be mainly classified into three categories: traditional time series prediction models, machine learning models, and hybrid models. One category is the traditional time series prediction models, such as the ARIMA model [11], SARIMA model [12], ARIMAX model [13], exponential smoothing(ES) model [14], and Markov switching model [15], which are the most widely used to forecast the incidence of human brucellosis. These traditional time series prediction models are based on a linear problem hypothesis [16] and have the advantages of easy modeling and simple calculations [17]. However, these models also have the disadvantage that they can only capture the linear relationship information in the time series, rather than dealing with nonlinear relationship information [18], resulting in some bias in making long-term forecasts for infectious disease prediction. The other category is machine learning models, represented by the XGBoost model [4], support vector machine (SVM) [19], multivariate adaptive regression splines (MARS) [19], random forest (RF) [20], and Elman and Jordan neural networks [21], Adaptive Neuro-Fuzzy Inference System (ANFIS) [22] have also been applied to forecast the incidence of human brucellosis. These models have the advantage of being proficient in addressing nonlinear problems. Nevertheless, these models also have some limitations. For example, the XGBoost algorithm is good at dealing with nonlinear data but has poor interpretability [18]. SVM is not excellent at handling a problem with several samples and variables, and while the Bayesian network can be trained quickly and efficiently, it lacks sufficient complexity [23]. The RF model cannot account for the specific nonlinear relationships between meteorological factors and diseases [20]. The last category is hybrid models, which include the ARIMA-BPNN [8], ARIMA-ERNN [8], and ARIMA-ETS [24] models, and are less commonly applied to human brucellosis incidence. The most advantageous feature of hybrid models is that they can fully exploit linear and nonlinear information in the time series to achieve better prediction results [8]. However, these models have special requirements for data samples and features, and whether they are suitable for other regions requires further study [25].

In 1944, Robert G. Brown proposed the exponential smoothing (ES) model, which is one of the most typical time series forecasting models for infectious diseases [26]. The TBATS model is a modification of the traditional ES model, and uses an exponential smoothing approach to solve complex periodic time series problems [27]. As a result, the TBATS model has the advantage of processing a wider variety of complex seasonal pattern time series [27] than the ES and ARIMA models. The structural components of the TBATS model mainly consist of trigonometric seasonality, Box-Cox transformation, ARMA errors, and trend and seasonal components [28]. The extreme learning machine (ELM), a new type of machine learning algorithm [29], was first proposed by Professor Guangbin Huang. The ELM model is a fast learning algorithm for a single hidden layer neural network [30]. The ELM model has been more widely used in other fields, such as industry [29], agriculture [31], the environment [32], geohazards [33], and medical care [34], but there are few reports on infectious disease prediction.

To date, there have been no reports on the use of TBATS and ELM models to predict cases of human brucellosis in China or worldwide. Therefore, in this study, the TBATS and ELM models were constructed based on monthly human brucellosis cases from January 2012 to December 2021 in 31 provinces and municipalities in mainland China and then compared with their prediction performance using MAE, MSE, MAPE, and RMSE indices. The most suitable model was chosen to forecast monthly human brucellosis cases from January to December 2022 in 31 provinces and municipalities in mainland China. To the best of our knowledge, this is the first study to develop TBATS and ELM models to forecast human brucellosis cases in mainland China. We hope that our research provides early warnings for effective prevention and control measures for human brucellosis and timely allocation of sufficient medical resources before this infectious outbreak in mainland China.

## Methods

### Data source

Data on monthly human brucellosis cases from January 2012 to December 2021 in 31 provinces and municipalities in mainland China were obtained from the National Health Commission of the People’s Republic of China website (http://www.nhc.gov.cn/) (Additional file 1). In China, the Law of the People’s Republic of China on the Prevention and Treatment of Infectious Diseases stipulates that infectious diseases are classified as Class A, Class B, and Class C with 40 types, and all medical institutions, Centers for Disease Control and Prevention (CDC), and blood collection and supply institutions are in charge of infectious disease reporting and management. Human brucellosis is classified as a Class B infectious disease under the Law of the People’s Republic of China on the Prevention and Treatment of Infectious Diseases. If a patient is diagnosed, the doctor must immediately report to the local health administration within 24 h. In this study, all human brucellosis cases were confirmed using laboratory tests and clinical diagnosis. Data on monthly human brucellosis cases were uninvolved with patients’ personal information; therefore, ethical approval was not required.

In our study, data from January 2012 to December 2021 with 120 observations spanning 10 years were collected, which met the requirements of the sample size and characteristics for TBTAS and ELM models construction. In addition, 120 observations were divided into the training and test sets. Data from January 2012 to December 2020 were used as the training set to construct the TBATS and ELM models, and data from January to December 2021 were used as the test set to evaluate the simulation prediction performance of each model.

### TBATS model

Traditional seasonal exponentially smooth models are limited in their ability to handle multi-seasonal, non-integer seasonal, and dual-calendar time series [27]. In this context, several researchers have studied the problem of processing complex seasonal time series, and the traditional exponentially smooth model was modified to resolve this problem [27].

The BATS model was introduced to address complex time series, such as multi-seasonal, non-integer seasonal, and dual-calendar time series [28]. The basic model form is expressed as BATS(p, q, m1, m2,..., mT), where B is the Box-Cox transformation, A is the ARMA error, and T and S are the trend and seasonal components of the time series, respectively [27]. In addition, parameters p and q of the BATS model are the ARIMA model orders p and q, and m1, m2,…, and mT are the seasonal periods of the ARIMA model. The mathematical formula for the BATS model is as follows [35].

1 $$y_{t}^{(\omega )} = \left\{ \begin{gathered} \frac{{y_{t}^{\omega } - 1}}{\omega }\begin{array}{*{20}c} {} & {\omega \ne 0} \\ \end{array} \hfill \\ \log y_{t} \begin{array}{*{20}c} {} & {\omega = 0} \\ \end{array} \hfill \\ \end{gathered} \right.$$

TBATS is a method for predicting time series data with the main goal of applying exponential smoothing to forecast complicated seasonal trends. The TBATS model, a modified time series approach based on the BATS model, was obtained by replacing the seasonal components with trigonometric seasonal functions [27, 28]. Therefore, the structure of this model’s initial T signifies the “trigonometric” [36]. This is expressed as TBATS(ω, p, q, $\varphi$, {m1, k1}, {m2, k2},…,{mT, kT}) [27]. The mathematical formula for the TBATS model is as follows [35].

2 $$y_{t}^{(\omega )} = \ell_{{\text{t - 1}}} + \varphi b_{t - 1} + \sum\limits_{i = 1}^{T} {s_{{t - m_{i} }}^{(i)} } + d_{t}$$

3 $$l_{t}^{{}} = \ell_{{\text{t - 1}}} + \varphi b_{t - 1} + \alpha d_{t}$$

4 $$b_{t}^{{}} = \varphi b_{t - 1} + \beta d_{t}$$

5 $$d_{t}^{{}} = \sum\limits_{i = 1}^{p} {\phi_{i} } d_{t - 1} + \sum\limits_{i = 1}^{q} {\theta_{i} } e_{t - 1} + e_{t}$$

Seasonal part can be written as:

6 $$s_{t}^{(i)} = \sum\limits_{j = 1}^{{(k_{i} )}} {s_{j,t}^{(i)} }$$

7 $$s_{j,t}^{(i)} = s_{j,t - 1}^{(i)} \cos (\omega_{i} ) + s_{j,t - 1}^{*(i)} \sin (\omega_{i} ) + \gamma_{1}^{(i)} d_{t}$$

8 $$s_{j,t}^{{{*}(i)}} = { - }s_{j,t - 1}^{(i)} \sin (\omega_{i} ) + s_{j,t - 1}^{*(i)} \cos (\omega_{i} ) + \gamma_{2}^{(i)} d_{t}$$

9 $$\omega = 2\pi j/m_{i}$$

where *ω* represents the Box-Cox transformation,$y_{t}^{{}}$ is the observation at time *t*, $\ell_{t}^{{}}$ is the local level in period *t,* $b_{t}^{{}}$ is the short-run trend in period *t*, $s_{t}^{(i)}$ is the seasonal component at time *t*, $d_{t}^{{}}$ is an ARMA(p,q) process, *m* and *T* are seasonal periods and *T* seasonal patterns, $\varphi$ is the dampening parameter value, m_i_ is the length of the ith seasonal period, *k* is the number of harmonics for the ith seasonal period, α and β are smoothing parameters, $\phi_{i}$ and $\theta_{i}$ are ARMA(p,q) coefficients,$e_{t}$ is Gaussian white noise, $\gamma_{1}$, and $\gamma_{2}$ are seasonal smoothing (two for each period) [35].

### ELM model

**Fig. 1:**
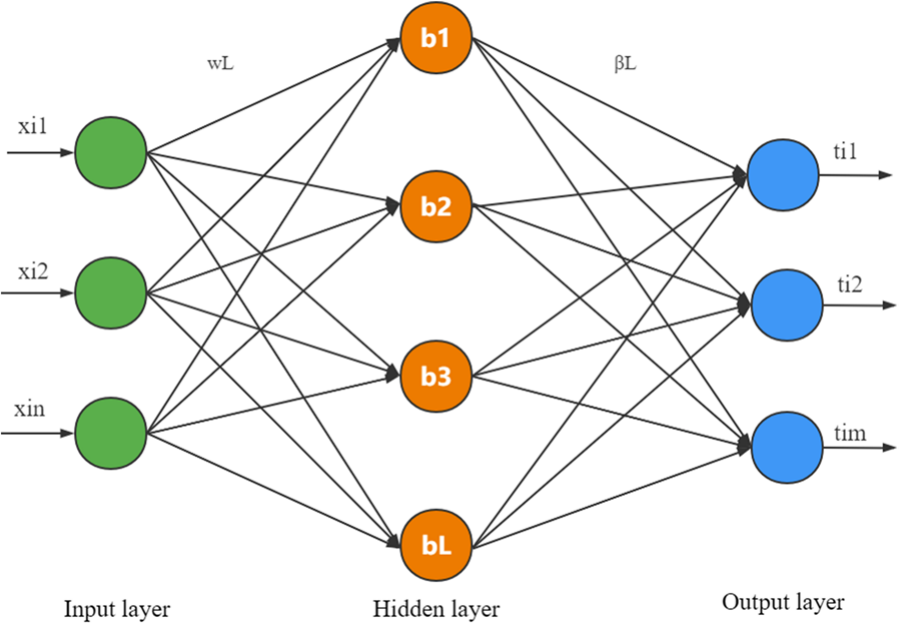
The network structure of ELM model

An extreme learning machine (ELM) was proposed by Guangbin Huang to solve regression and classification issues [37]. ELM is a single hidden layer feedforward neural network (SLFNs) algorithm [30], and its model structure consists of three layers: input layer, hidden layer, and output layer (Fig. 1). Compared with traditional machine learning models, the ELM model has two prominent characteristics [30]: (1) the connection weights between the input layer and the hidden layer, and the threshold value of the hidden layer can be set randomly and no adjustment is required after setting; (2) the output layer weight can be solved as the least squares problem. Therefore, the ELM model has a faster learning speed and better generalization performance than traditional machine learning algorithms under the premise of ensuring learning accuracy.

The ELM model learning principles and the main algorithm steps are as follows:

Assume that there is an arbitrary sample N(xi, ti), xi is the input vector, xi = [xi, xi,2,..., xi,n] ∈ R^n^, ti is the target, ti = [ti, ti,2,..., ti,m] ∈ R^m^, and for an ELM network with L hidden layer nodes can be expressed as [29]:

10 $${\text{O}}_{i} = \sum\limits_{k = 1}^{L} {\beta_{k} } g_{{}} (x_{i} \cdot w_{k} + b_{k} );\begin{array}{*{20}c} {} & {i = 1,2,...N;} \\ \end{array}$$

where Oi is the output value, *g*(x) is the activation function, w_i_ is the input weight (w_i_ = [*w*_*1*_*, w*_*2*_*,..., w*_*L*_]), b is the bias vector (b = [*b*_*1*_*, b*_*2*_*,..., b*_*L*_]), β is the output weight (β = [*β1, β2,..., βL*]), and $x_{i} \cdot w_{k}$ is the inner product.

Equation (3) can be rewritten as O = Hβ, where O is the expected output and H is the output of the hidden layer matrix, which can be expressed as [38]:

11 $${\text{H}} = \left[ \begin{gathered} g(x_{1} \cdot w_{1} + b_{1} )...g(x_{1} \cdot w_{L} + b_{L} ) \hfill \\ \begin{array}{*{20}c} {\begin{array}{*{20}c} {\begin{array}{*{20}c} {\begin{array}{*{20}c} {} & \vdots & {} \\ \end{array} } & {} & {} \\ \end{array} } & {} & {} \\ \end{array} } & \vdots & {} \\ \end{array} \hfill \\ g(x_{N} \cdot w_{1} + b_{1} )...g(x_{N} \cdot w_{L} + b_{L} ) \hfill \\ \end{gathered} \right]_{{{\text{N}} \times {\text{L}}}}$$

The goal of training is to obtain the training error and output weights with the smallest norm, which can be expressed as

12 $$\min \left\| {O - T} \right\|^{2} = \sum\limits_{i = 1}^{N} {\left\| {o_{i} - t_{i} } \right\|^{2} }$$

where T denotes the target matrix. The least-squares method for the solution was then applied to calculate, as follows:

13 $$\beta = {\text{H}}^{ + } {\text{T}}$$

where β is the output weight and H^+^ is the generalized inverse matrix of H, which can be calculated as H^+^ = (H^T^H)^−1^H^T^ [38].

### Evaluation of prediction performance

The mean absolute error(MAE), mean squared error(MSE), mean absolute percentage error(MAPE), and root mean square error (RMSE) were calculated to evaluate the prediction accuracies of the TBATS and ELM models. The smaller the values of MAE, MSE, MAPE, and RMSE, the better is the prediction performance of the model [8]. Generally, a fitted model with a MAPE value lower than 0.2 [39] indicates that this model has superior predictive performance. The indicators are expressed as follows:

14 $${\text{MAE}} = \frac{{\sum\limits_{t = 1}^{n} {\left| {X_{t} - {\hat{\text{X}}}_{t} } \right|} }}{n}$$

15 $${\text{MSE}} = \frac{1}{n}\sqrt {\sum\limits_{t = 1}^{n} {(X_{t} - \hat{X}_{t} )^{2} } }$$

16 $${\text{MAPE}} = \frac{{\sum\limits_{t = 1}^{n} {\left| {\frac{{X_{t} - {\hat{\text{X}}}_{t} }}{{X_{t} }}} \right| \times 100{\text{\% }}} }}{n}$$

17 $${\text{RMSE}} = \sqrt {\frac{{\sum\limits_{t = 1}^{n} {(X_{t} - {\hat{\text{X}}}_{t} )^{2} } }}{n}}$$

where ${\hat{\text{X}}}_{t}$ is the predicted value, $X_{t}$ is the observed value of monthly human brucellosis cases, and n is the sequence sample size.

### Data analysis

The R software version 4.1.1 was applied to construct the TBATS model, and the “forecast,” “zoo,”and “tseries” packages were used in the construction of the TBATS model. The MATLAB software (Version R2020b, MathWorks, Natick, MA, USA) was used to construct the ELM model. The level of significance was set at *p* < 0.05.

## Results

### General description

**Fig. 2:**
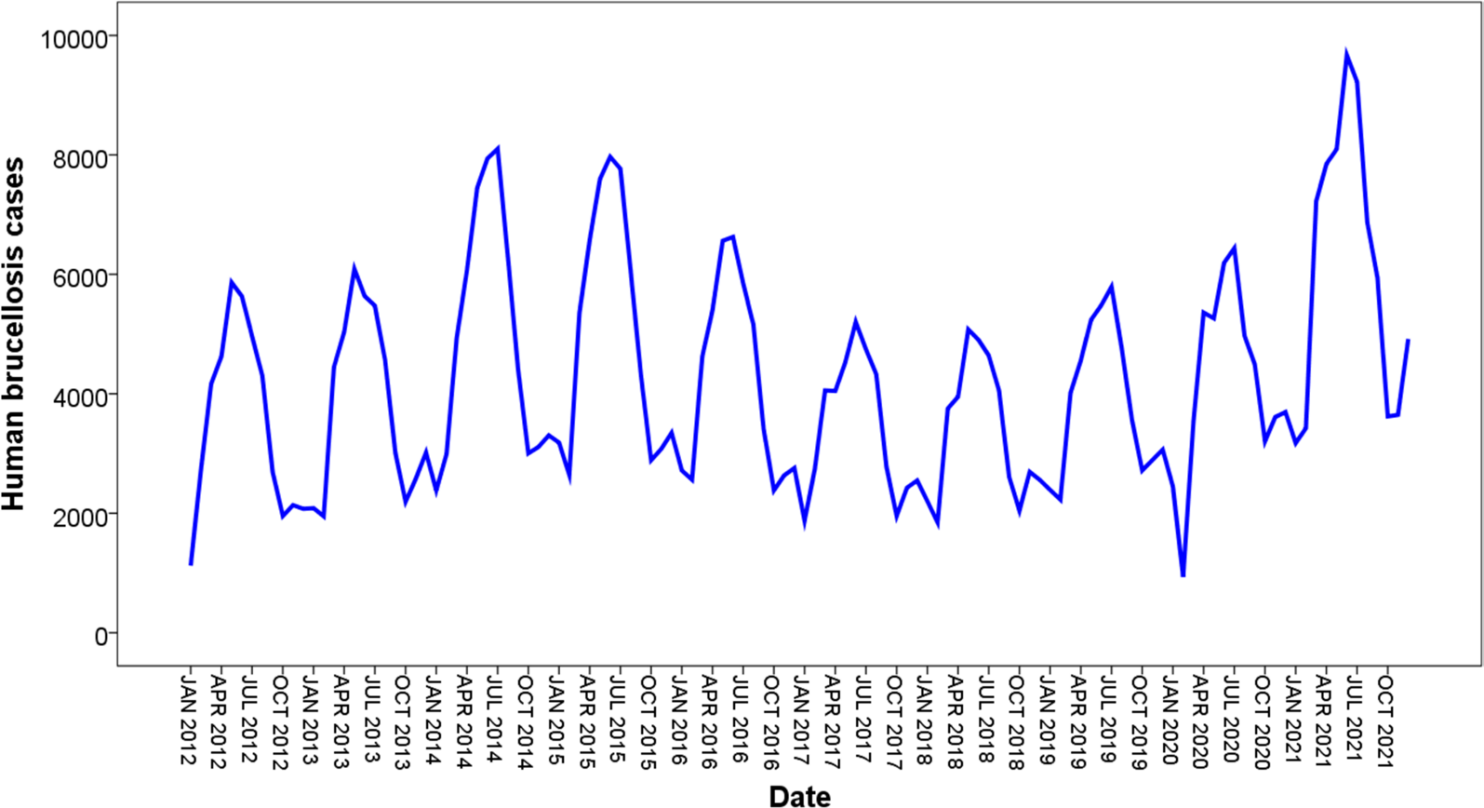
The diagram of original time series from human brucellosis cases from January 2012 to December 2021

**Fig. 3:**
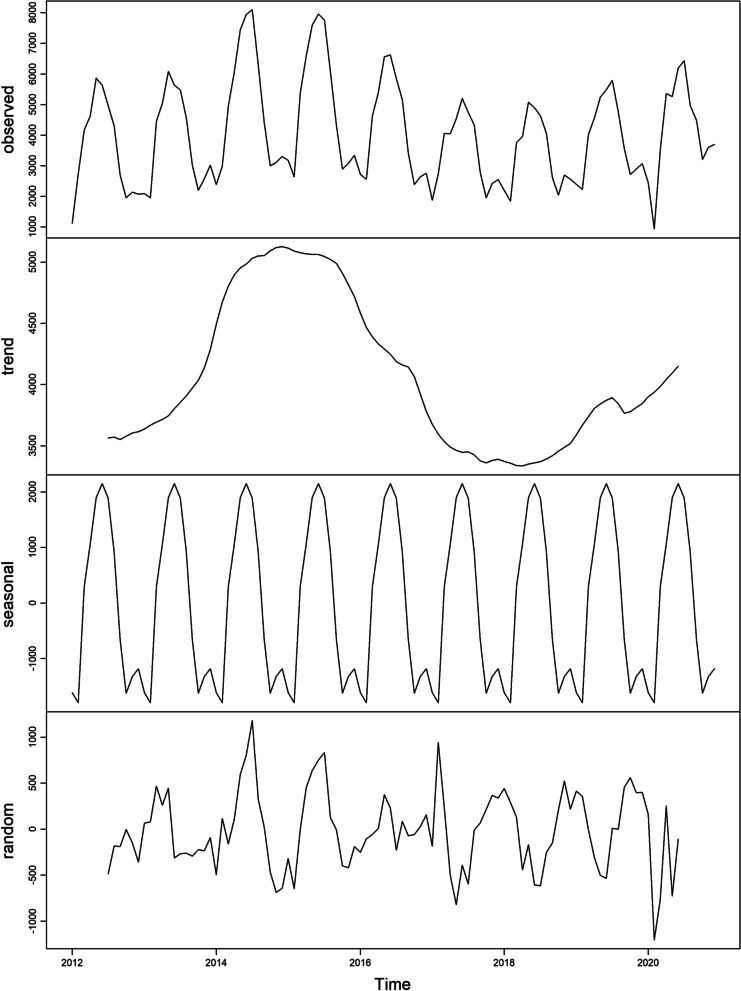
Seasonal decomposition of the monthly human brucellosis cases from January 2012 to December 2021 in mainland China. From top to bottom, in the following order observed, trend, seasonal, and random of the human brucellosis cases time-series

A total of 511,826 human brucellosis cases were reported from January 2012 to December 2021 in 31 provinces and municipalities in mainland China. As shown in Figs. 2, 3, human brucellosis cases exhibit obvious seasonality, cyclicality, trends, and randomness, with the highest peak from April to May and the lowest peak from September to October of each year.

### TBATS model

The accuracy prediction performance of the TBATS model depends to a high degree on the number of harmonics, k, for the seasonal component. The optimal TBATS model not only requires adjustment of the number of harmonics k values and keeping all other harmonics constant for each seasonal component with the lowest AIC value but also requires suitable parameters p and q of the ARMA model [27].

**Fig. 4:**
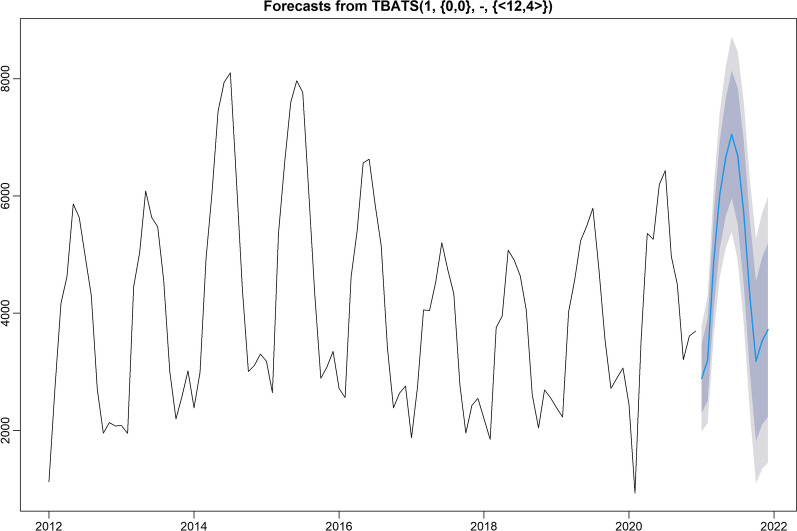
Forecasts of the monthly human brucellosis cases from the TBATS model. Validation set part: the solid blue line is the predicted value, the lavender area is the 80% confidence interval for the predicted value, and the light grey area is the 95% confidence interval of the predicted value

The tbats() function of R software was applied to automatically build the TBATS model. The optimal model was TBATS(1, {0,0}, -, {< 12,4 >}), and the parameters $\omega$, p, q, k, and the Fourier terms were 1, 0, 0, 12, and 4, respectively. Furthermore, the parameters of this model are that the smoothing parameter $\alpha$ is 0.6909, the seasonal smoothing parameters $\gamma_{1}$ and $\gamma_{2}$ are − 0.010 and 0.005, $\sigma^{2}$ is 461.4664, and the lowest value of AIC is 1854.703. Therefore, the optimal TBATS(1, {0,0}, -, {< 12,4 >}) model was used to forecast monthly human brucellosis cases from January to December 2021 in 31 provinces and municipalities of mainland China. The results are presented in Tables 1 and Fig. 4, respectively.

**Table 1:** Forecasts of the monthly human brucellosis cases from January to December 2021

Date	Actual values	TBATS	ELM
21-Jan	67,682	3175	3477
21-Feb	44,933	3425	2659
21-Mar	73,427	7220	4440
21-Apr	85,684	7848	7398
21-May	83,385	8096	8827
21-Jun	84,952	9670	8569
21-Jul	83,101	9222	9334
21-Aug	76,423	6867	6844
21-Sep	75,409	5932	3737
21-Oct	67,843	3622	3408
21-Nov	69,640	3649	3147
21-Dec	64,097	4919	2441

### ELM model

**Fig. 5:**
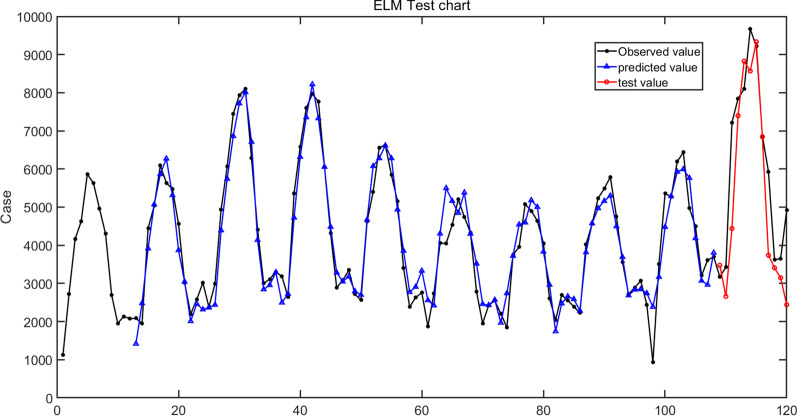
Forecasts of the monthly human brucellosis cases from the ELM model. The solid black line is the observed value, the solid blue line is the predicted value, and the solid red line is the validation value from January to December 2021

We used an ELM model, which included an input layer, a single hidden layer, and an output layer, and applied it to solve regression issues. First, we used a sliding window algorithm approach to determine the input and output variables, and the window was set to 12; therefore, 12 input variables and 1 output variable were determined. The input and output data were normalized before modeling. Second, through repeated experiments, the suitable number of hidden layers was found to be 33, the R-squared was 0.89, the activation function used the sigmoid function, and the optimal ELM model was constructed. Finally, the optimal ELM model was applied to forecast monthly human brucellosis cases from January to December 2021 in 31 provinces and municipalities in mainland China. The results are shown in Table 1 and Fig. 5.

### Prediction performance

**Fig. 6:**
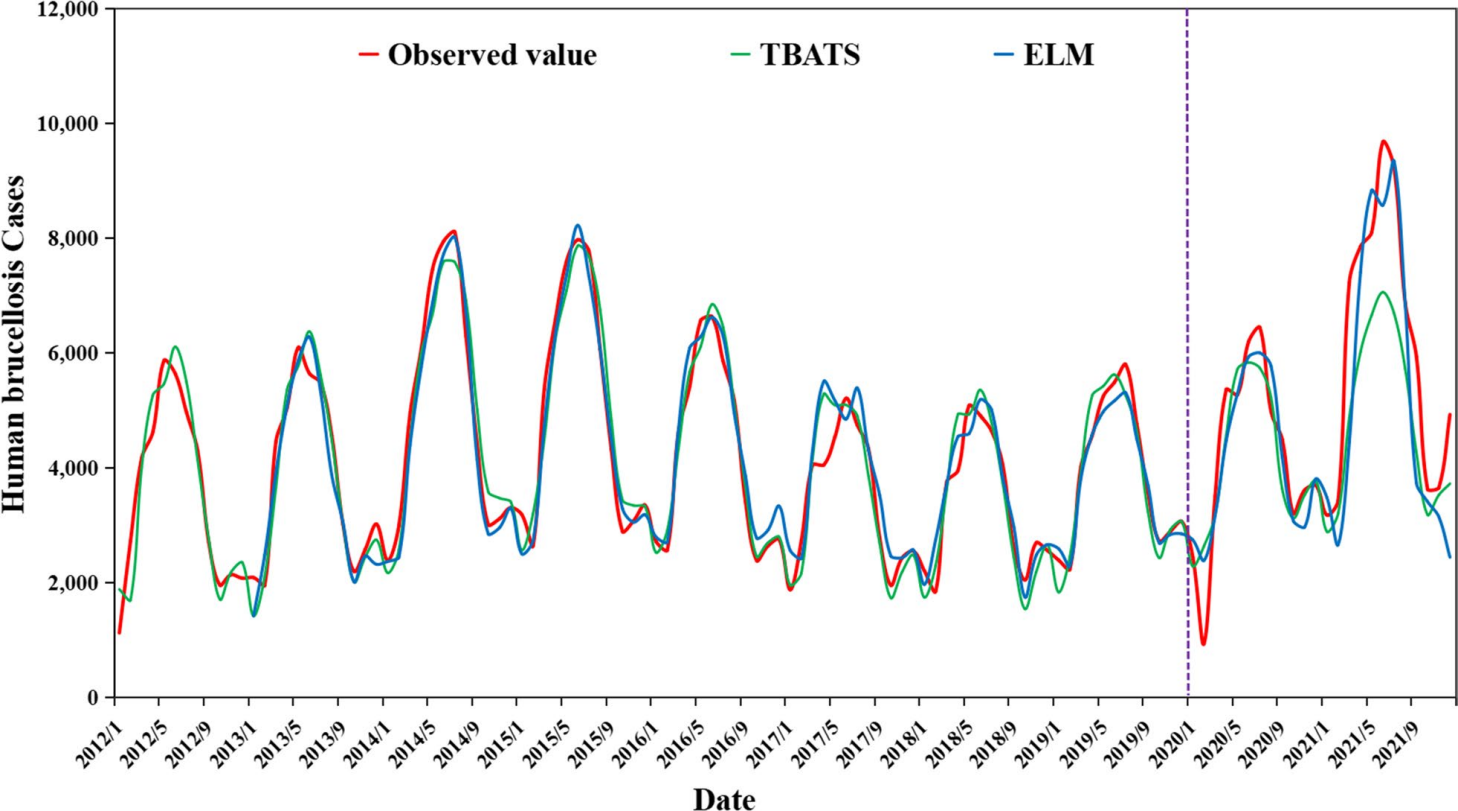
The predictive values fitted by ELM and TBTAS models. The red solid line is the observed value, the solid green line is the predicted value fitted by the TBTAS model, and the solid blue line is the predicted value fitted by the ELM model

During the ELM modeling process, a sliding window algorithm approach was used to identify the input and output variables; therefore, 12 predicted values were lost in the modeling process, resulting in 108 predicted values for the evaluation of the prediction performance. Measure indices, including MAE, MSE, MAPE, and RMSE, were used to evaluate the prediction performances of the TBATS and ELM models. The results showed that the MAPE values of the ELM and TBATS models were both lower than 0.2, suggesting that they have superior predictive performance and can be used to forecast monthly human brucellosis cases. However, the ELM model is the optimal model because it has the lowest MAE, MSE, MAPE, and RMSE values for both fitting and forecasting performance (Table 2). As shown in Fig. 6, the predictive values fitted by the ELM model better simulated the trend of the actual situation changes in monthly human brucellosis cases in mainland China. In particular, in terms of forecasting performance, the values of MAE, MSE, and MAPE were significantly lower than those of the TBTAS model, indicating that ELM has a stronger generalization ability.

**Table 2:** Comparison of forecasts of ELM and TBTAS models in fitting performance and forecasting performance

Evaluating indices	Fitting performance		Forecasting performance	
	ELM	TBTAS	ELM	TBTAS
MAE	337.51	355.85	971.17	1322.92
MSE	2151.85	2241.68	2325.12	2745.05
MAPE(%)	10.60	10.80	17.10	19.10
RMSE	3043.17	3170.21	3288.22	3882.08

### Simulation prediction results

The trained ELM model was applied to forecast monthly human brucellosis cases from January to December 2022 in 31 provinces and municipalities of mainland China. Table 3 presents the results.

**Table 3:** The forecasts of the monthly human brucellosis cases in mainland China 2022

Date	Predicted values	Date	Predicted values
22-Jan	2686	22-Jul	4230
22-Feb	2489	22-Aug	3048
22-Mar	3504	22-Sep	3334
22-Apr	6407	22-Oct	2611
22-May	5907	22-Nov	3157
22-Jun	5575	22-Dec	5627

## Discussion

Human brucellosis has re-emerged in China since the early twenty-first century [40]. In the past few decades, peasants and herders have been prone to brucellosis in northern, northeastern, and western China [2, 40]. However, human brucellosis cases were found in urban workers and food service workers in southern China because of their eating habits or travel to or from endemic areas [41]. Currently, the prevalence and transmission of human brucellosis continues to outbreak in both the south and north of mainland China among peasants, herdsmen, and urban workers; therefore, human brucellosis prevention and control remains an important task for public health prioritization in mainland China.

Our findings showed that the incidence of human brucellosis has increased from January 2012 to December 2021 in mainland China. This may be because of several reasons. First, with rapid economic development in China, both in urban and rural areas, residents’ demand for meat is gradually increasing, especially for mutton and beef [41]. Research has shown that sheep are a major factor in the spread of human brucellosis [42]. As a result, the increased demand for mutton and beef by residents has led to the rapid development of animal husbandry and increased demand for meat importation in China [42], thus leading to an increased risk of exposure to live or slaughtered meat. Second, occupation is also an important factor affecting the incidence of brucellosis [43]. Studies have also shown that slaughterhouse workers, meat-packing employees, veterinarians, and herdsmen comprise the majority of brucellosis patients [42]. Li et al. [44] investigated the understanding of brucellosis prevention and control among peasants and herders in Mubi County, Xinjiang, China. Their study showed that the general knowledge of brucellosis among peasants and herders in a township in Mubi County was 71.9%, and the knowledge of brucellosis prevention and control was only 48.3%. The results of this study showed that peasants and herdsmen in western China still lack knowledge of brucellosis prevention and control, which not only makes brucellosis prevention difficult but also increases the risk of developing brucellosis.

The results also showed that the incidence of brucellosis displayed significant seasonal characteristics between 2012 and 2021, with the highest peak in early spring to early summer, and the lowest peak in winter, consistent with previous studies [8–10, 14, 24, 40, 42]. This might be related to meteorological and economic factors in animal husbandry [8]. Peng et al. [42] found that mid-temperate and warm-temperate climates strongly affect the emergence of brucellosis. Moreover, mid-temperate and warm-temperate climates are characterized by high temperatures in summer and cold, dry winters. Relatively low temperatures and humidity in autumn and winter reduce the survival rate of pathogenic bacteria, thereby reducing the chance of infection in humans [8, 45]. Therefore, the incidence of brucellosis was higher in spring and summer than in fall and winter. Animal husbandry is also an important factor in the occurrence of brucellosis [8]. The increasing number of sheep has led to a high incidence of brucellosis in China [42]. In spring, as herders engage in more farming activities, there is closer contact with sheep, such as shearing sheep and cleaning up sheep manure, increasing the risk of transmission of brucellosis [8, 42]. Summer is the peak delivery season for livestock, such as cattle and sheep, which greatly increases the chances of exposure to pathogenic factors during this process [46]. Furthermore, because lambs are born in winter or early spring, peasants and herders are at an increased risk of brucellosis infection from contact with amniotic fluid or infected young animals [40].

In real-world disease surveillance scenarios, the data of infectious disease time series generally display non-stationary and complex characteristics [4]. Therefore, appropriate models should be selected to predict infectious diseases based on the distribution and characteristics of data. In this study, we established ELM and TBATS models based on sample size and data characteristics. Our results also showed that both ELM and TBATS models could be used to predict the incidence of human brucellosis; however, the prediction performance of the ELM model outperformed that of the TBATS model. There are several possible reasons for this finding. First, although the TBATS model can handle complex periodic time series [27, 28], it is based on a mixture of multiple time series forecasting methods. The computation is more complicated, and if the model is not updated in time, the accuracy of the prediction may be reduced [27]. Second, in recent years, machine learning predictive models have been increasingly applied to infectious disease prediction and have achieved satisfactory prediction performance. ELM is a type of machine learning model [30] that has several advantages over traditional machine learning models [30]. By randomly generating the weight and bias of the hidden layer [30, 47], the ELM model exhibits excellent generalization and faster learning speed [33]. Moreover, the ELM model is easy to develop and achieves the smallest training error and smallest norm of weights [38]. Third, the optimal ELM model requires repeated trials and constant adjustment of model parameters to achieve better prediction results. In our study, through repeated experiments, the optimal ELM model was determined by obtaining the maximum R-squared value and the minimum MAPE and RMSE values under continuous adjustment of the number of hidden layers. When the hidden layer is adjusted to 33, the R-squared value is at a maximum, whereas the MAE and MAPE values are at a minimum, and then the optimal ELM model is obtained.

Our study demonstrated that the ELM model is more suitable for predicting human brucellosis cases in mainland China; that is, the prediction performance of machine learning models is better than that of time series models, which is in agreement with previous research [4, 19, 21]. Alim et al.[4] compared the ARIMA and XGBoost models for the prediction of human brucellosis in mainland China, and their study indicated that the prediction performance of the XGBoost model outperformed the ARIMA model. Bagheri et al. [19] used a support vector machine (SVM), multivariate adaptive regression splines (MARS), random forest (RF), and ARIMA models to predict monthly brucellosis cases in Iran. Their study showed that the MARS model was more appropriate than other models. Wu et al. [21] constructed a seasonal ARIMA model and Elman and Jordan recurrent neural networks to forecast the incidence of human brucellosis in mainland China, and the result showed that the Elman and Jordan recurrent neural networks achieved better prediction performance than ARIMA model. However, some studies have reported the opposite. For example, Zheng et al. [12] indicated that the forecasting performance of the SARIMA model was better than that of the NARNN model for predicting the incidence of human brucellosis in Xinjiang, China. This contradictory conclusion may be caused by the different sample sizes, data characteristics of the time series, and research at different study sites.

Our study had some limitations. First, although the ELM model has achieved superior predictive performance compared to TBATS models in forecasting monthly human brucellosis cases in mainland China, it can also be significantly influenced by the random selection of input weights and SLFN biases [30]. Second, the occurrence and prevalence of brucellosis are influenced by multiple factors [4], for example, meteorological, environmental, and medical factors. However, factors that influenced the occurrence and prevalence of brucellosis were excluded from the forecasting models. Consequently, there was some bias in the prediction results of our study.

## Conclusions

Our study showed that the ELM model had a better prediction performance than the TBATS model in predicting human brucellosis cases. These prediction results can provide information for the prevention, control, and monitoring of human brucellosis in mainland China. Furthermore, the incidence of human brucellosis exhibited seasonal characteristics, the government and relevant departments should develop appropriate preventive measures in early spring and early summer, establish a joint prevention and control mechanism for major zoonotic diseases, and at the same time, increase investment funds to train peasants and herdsmen and other relevant personnel on brucellosis epidemic prevention.

## Supplementary Information

**Additional file 1. **Data on monthly human brucellosis cases from January 2012 to December 2021 in 31 provinces and municipalities in mainland China.

## Data Availability

Data supporting the findings of this study are available from the National Health Commission of the People’s Republic of China website (http://www.nhc.gov.cn/) without restrictions.

## Abbreviations

TBATS: Trigonometric seasonality, Box-Cox transformation, ARMA errors, trend, and seasonal components
ELM: Extreme learning machine
ARIMA: Autoregressive integrated moving average
SARIMA: Seasonal autoregressive integrated moving average
ES: Exponential smoothing
XGBoost: Extreme gradient boosting
SVM: Support vector machine
SVR: Support vector regression
MARS: Multivariate adaptive regression splines
RF: Random forest
BPNN: Back propagation neural network
ERNN: Efficient recurrent neural networks
ETS: Error, trend, seasonality
LSTM: Short-term memory
ANNs: Artificial neural networks
ANFIS: Adaptive Neuro-Fuzzy Inference System
MAE: Mean absolute error
MSE: Mean square error
MAPE: Mean absolute percentage error
RMSE: Root mean square error
AIC: Akaike information criterion
